# Cell wall remodeling under abiotic stress

**DOI:** 10.3389/fpls.2014.00771

**Published:** 2015-01-07

**Authors:** Raimund Tenhaken

**Affiliations:** Department of Cell Biology, Plant Physiology, University of SalzburgSalzburg, Austria

**Keywords:** abiotic stress, peroxidase, xyloglucan endotransglucosylases/hydrolases, reactive oxygen species, boron

## Abstract

Plants exposed to abiotic stress respond to unfavorable conditions on multiple levels. One challenge under drought stress is to reduce shoot growth while maintaining root growth, a process requiring differential cell wall synthesis and remodeling. Key players in this process are the formation of reactive oxygen species (ROS) and peroxidases, which initially cross-link phenolic compounds and glycoproteins of the cell walls causing stiffening. The function of ROS shifts after having converted all the peroxidase substrates in the cell wall. If ROS-levels remain high during prolonged stress, OH°-radicals are formed which lead to polymer cleavage. In concert with xyloglucan modifying enzymes and expansins, the resulting cell wall loosening allows further growth of stressed organs.

## Introduction

Abiotic stress in plants refers to growth conditions which are unfavorable for growth. Traditionally, these conditions include temperature stress (either cold or heat) as well as drought, osmotic stress and salinity. The latter stress factors are tightly associated with scarce water resources, a major challenge for future farming given the predicted increase of human population in the next decades (Rosegrant and Cline, 2003). Furthermore, an imbalance in nutrients (e.g., boron) in the soil can be an important abiotic stress factor affecting plant cell walls (Wimmer and Eichert, 2013). In nature, plants may be exposed to several stress conditions at the same time (Mittler and Blumwald, 2010). Atkinson and Urwin (2012) found evidence that the plant response to multiple environmental stresses is distinct from that of individual stress factors and not just additive.

Cell wall dynamics is not easy to analyze and as a consequence the majority of studies about cell wall modifications under abiotic stress focus primarily on genes putatively involved in cell wall metabolism. The improvement of proteomics technologies has further led to studies in which the plant proteome is compared between stressed and control plants including a few studies focusing on the cell wall proteome (Dani et al., 2005; Zhu et al., 2007; Kong et al., 2010; Shinano et al., 2011; Komatsu and Yanagawa, 2013). The changes in the cell wall itself are only barely studied.

Most plants stop growing upon exposure to severe drought, osmotic stress or salt stress (Skirycz and Inze, 2010). In aerial parts of plants the cessation of growth usually occurs earlier than in the root system (Wu and Cosgrove, 2000) allowing the stressed plants to invest available resources into root growth to explore residual water in the soil. Growth of plant organs is based on cell division in the meristematic zones followed by a tremendous expansion of new cells in a complex turgor driven process (Schopfer, 2006). Therefore, it is obvious that any reduction in cell turgor, caused by osmotic stress, also reduces the mechanical power of the cell to expand the polysaccharide network.

### The plant cell wall

Plant cell walls are composed of carbohydrate polymers, lignin and structural proteins in variable amounts. The cell wall is of critical importance for the cell shape and provides mechanical strength to withstand the turgor pressure. Furthermore, the cell wall is the front line for attacking pathogens. In primary cell walls all these functions need to be compatible with the expansion of cells during plant growth. Numerous excellent reviews about cell wall biology have been published over the years (Carpita and Gibeaut, 1993; Cosgrove, 1997; Reiter, 2002; Wolf et al., 2012; Braidwood et al., 2014). Many models of plant cell walls have been described which try to integrate all compositional and structural details which have emerged from the many studies on plant cell walls. Certainly there is no universal model which applies for all cell walls but a number of common principles have been discovered which are present and important in larger groups of plants. Primary cell walls consist of cellulose, a diverse group of different hemicelluloses binding to cellulose and pectins. The cellulose content in primary cell walls is rather low (often below 20%) but it provides mechanical strength for load bearing. In order to fulfill this function, the cellulose fibrils need to be cross-linked by hemicelluloses (Cosgrove, 1997; Wolf et al., 2012) and possibly also by pectins (Mohnen, 2008; Peaucelle et al., 2012). Xyloglucans are the major hemicellulosic polymers of dicot plants that bind to cellulose fibrils. Existing models suggest the binding of each xyloglucan polymers to at least two cellulose fibers (Cosgrove and Jarvis, 2012). This interaction can be modulated by two groups of enzymes, expansins and xyloglucan endotransglucosylases/hydrolases (XTH). The major group of polymers in primary dicot cell walls are pectins, a heterogeneous group of homogalacturonic acid, rhamnogalacturonan I (RG-I) and rhamnogalacturonan II (RG-II) (Mohnen, 2008). Homogalacturonic acid is a polymer of galacturonic acid, ionically cross-linked by Ca^++^, when the carboxygroup is not methylated. RG-I has a heteropolymeric backbone with alternating rhamnose and galacturonic acid molecules. An important feature of RG-I are the decorating side chains of arabinan or galactan (Mohnen, 2008). RG-II is a highly complicated polymer with a polygalacturonic backbone and four distinct side chains. Initially, pectins were mostly regarded as filling material for the open space in the cellulose /hemicellose network but newer studies point to important structural roles (Peaucelle et al., 2012). One of the oldest observations for this is the role of boron for cell walls (Lee and Aronoff, 1966) (see below), which is crosslinking pectic RG-II-molecules (Ishii et al., 1999). Without proper RG-II-interaction, the cell walls swell and increase in thickness concomitant with an increase in pore size (Fleischer et al., 1999).

## Peroxidases

Cell walls contain a number of structural proteins, as well as hemicellulosic polymers with attached phenolic residues like ferulated arabinoxylans, which can be cross-linked by the action of cell wall peroxidases (Lindsay and Fry, 2008; Burr and Fry, 2009). A number of studies have found peroxidases to be induced by osmotic stress (Simonovicova et al., 2004; Csiszar et al., 2008, 2012; Pechanova et al., 2010; Ranjan et al., 2012; Maia et al., 2013). By comparing wheat cultivars with different drought tolerance Csiszar et al. (2012) found higher transcripts levels for *TaPrx01, TaPrx03, TaPrx04* cell wall bound peroxidases in the drought tolerant variety when exposed to osmotic stress. The total peroxidase enzyme activity for covalently bound peroxidases under osmotic stress, using guaiacol as a substrate, was however higher in drought sensitive cultivar. A similar increase in peroxidase in cowpea was attributed to the inhibition of root growth under salinity (Maia et al., 2013), whereas a moderate dehydration did neither increase the cell wall bound peroxidase activity nor the root growth. A study performed with cotton roots found high peroxidase transcript levels mainly in two drought tolerant cultivars (Ranjan et al., 2012) whereas these genes are much lower expressed in two drought sensitive lines. A comparison of two wheat cultivars differing in their drought stress resistance also showed an increase in transcript levels of several peroxidases in the more resistant cultivar (Secenji et al., 2010). It was assumed that peroxidase in this system is more involved in scavenging hydrogen peroxide rather than in modifying the cell wall. The sensitive cultivar shows higher transcript levels for glutathione-S-transferases, which might be less efficient in removing reactive oxygen species. In a search for peroxidase genes from sweet potato Kim et al. (2008) identified one isoform (*SWPA4*) that is strongly induced by several abiotic stresses. They overexpressed the gene in tobacco plants and found a highly elevated peroxidase enzyme activity in the cell wall leading to transgenic plants which are more tolerant against salt and drought stress.

Peroxidases need H_2_O_2_, a common reactive oxygen species (ROS) in plants, as a co-substrate. ROS have been observed to be a typical plant response to osmotic stress (Miller et al., 2010) thereby matching the demand for the co-substrate of peroxidases. If both, the increase in peroxidase activity and the formation of ROS, occurs in the same tissue, cross-linking of cell wall components might strengthen the mechanical properties of the wall (Kieffer et al., 2000; Passardi et al., 2005; Wakabayashi et al., 2012). Cross-linking likely occurs between cell wall glycoproteins but also between phenolic compounds present in the wall in particular ferulic acid esterified to arabinoxylans in grasses (Lindsay and Fry, 2008; Burr and Fry, 2009). As this type of cross-linking is based on covalent linkages it can usually not been reversed. A more or less local stiffening of the cellular structure has occurred which is accompanied with a cessation of growth (MacAdam and Grabber, 2002; Wakabayashi et al., 2012; Uddin et al., 2014). This might be beneficial for plant cells to better withstand the changes in turgor pressure of osmotic stress. As discussed below the loosening of cell wall polysaccharides seems to be very important under osmotic, drought or salt stress to maintain the possibility for cells and organs to expand. The other face of ROS is the formation of OH° radicals, which are capable of cleaving sugar bonds in plant polysaccharides (Fry, 1998; Schopfer, 2001; Renew et al., 2005). This causes a cell wall loosening similar to the action of classical loosening enzymes like expansins or xyloglucan modifying enzymes (Renew et al., 2005).

The apparently contradicting observations about peroxidase activity under drought stress should be examined in broader sense, which considers all, peroxidase activity, substrates and ROS as a unit. The balance between these factors may well explain the different results observed in plant studies with abiotic stress. An excess of peroxidase activity, cross-linkable substrates and sufficient amounts of H_2_O_2_ will favor the local stiffening of the wall, reduce cell wall expansion and thus strengthen the mechanical stability of the cells and organs. If however H_2_O_2_ transiently accumulates in the presence of copper or iron in the cell wall, which are typically present in cell walls in sufficient amounts, and the peroxidase activity or the substrate availability is limited, the formation of OH°-radicals will be favored. This will cause disruption of sugar polymers of the cell wall with a concomitant weakening in the mechanical properties of the wall (Fry, 1998).

## Xyloglucan modification

**Figure 1:**
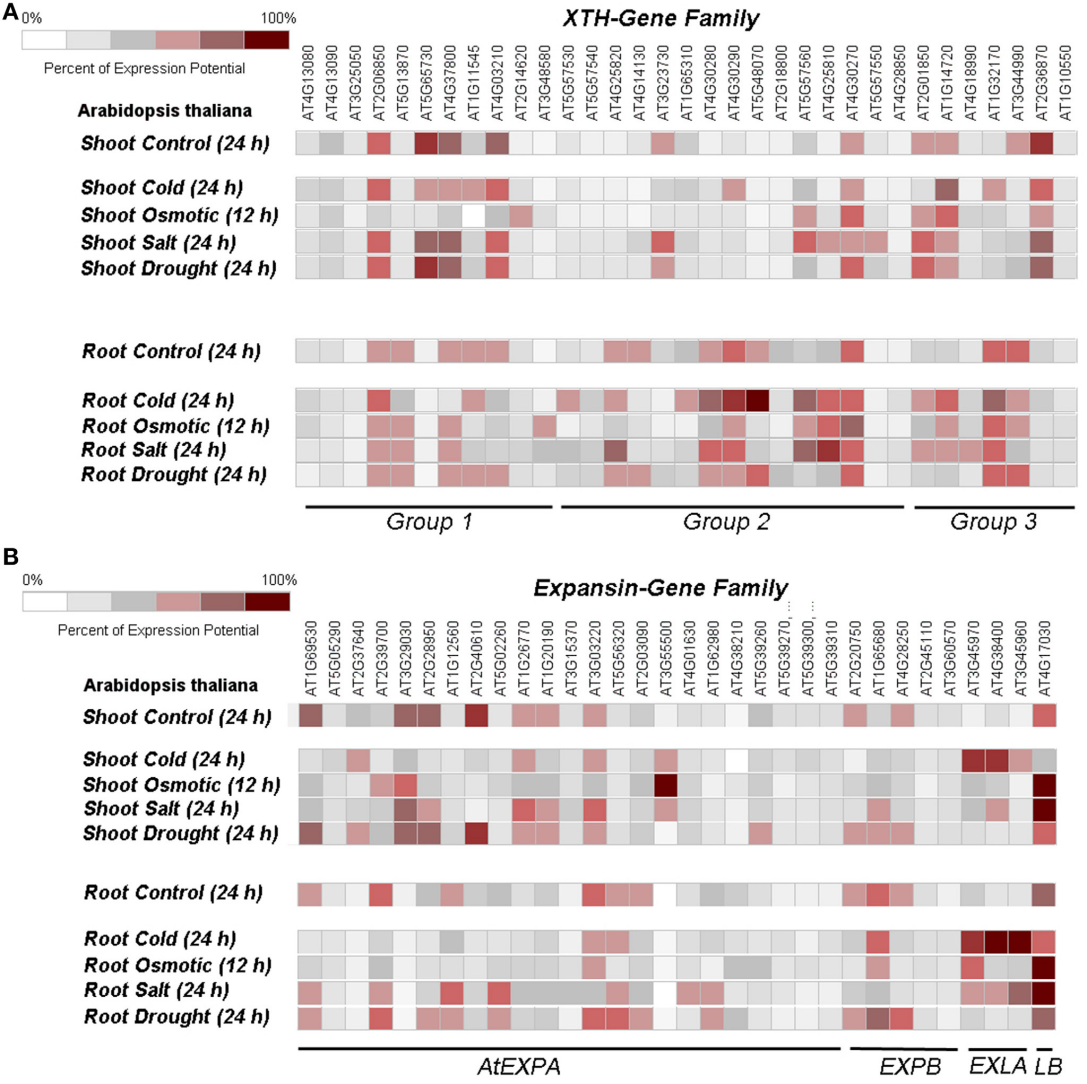
**Heat map of expansin and XTH gene expression under abiotic stress for (A) XTH gene family and (B) expansin gene family**. The XTH gene family is classified according to Rose et al. (2002), the expansins are classified according to Lee et al. (2001). Most but not all members are present on the Affimetrix ATH1 chip. The microarrays from the Atgenexpress dataset were used and visualized using the GENEVESTIGATOR software Kilian et al. (2007).

The xyloglucan endotransglucosylases/hydrolases (XTH) mediated cell wall remodeling is based on a widely proposed cell wall model, in which xyloglucans act as tethering polymers between load bearing cellulose fibrils (Hayashi, 1989). The most striking cell wall related phenotype observed in osmotically stressed plant cells is the increased expression of expansins and/or xyloglucan modifying enzymes (XTH) (Rose et al., 2002). Cho et al. (2006) have expressed a XTH gene from *Capsicum annuum* (*CaXTH*) constitutively in Arabidopsis, which was previously identified as an abiotic stress (cold, drought, salt) induced gene in hot pepper. The strong reduction in root length observed in control seedlings of Arabidopsis on salt-containing media is less pronounced if the *CaXTH* gene is expressed. However, aberrant leaf morphology (curled leaves) was found in several independent lines and transverse sections of leaves showed an irregular cell pattern compared to wild type plants. The same *CaXTH* gene was later expressed in tomato plants without phenotypical side effects (Choi et al., 2011). Again the transgenic tomato seedlings showed a strongly increased salt tolerance with longer roots. The exact mode of action was not determined but the authors speculate about a beneficial cell wall strengthening of mesophyll cells, protecting them from excessive water loss. In addition, they suggest an important role of the XTH activity in remodeling the cell wall of stomata possibly preventing excess water loss. Arabidopsis plants with a reduced level of cytokinin are more salt tolerant than the wild type. A transcriptome profiling identified numerous genes upregulated in the tolerant mutant, among them XTH genes and glycoside hydrolases (Nishiyama et al., 2012). Rice plants exposed to abiotic stress (cold, heat, drought) showed a strong increase in transcripts for *OsXET9*, a xyloglucan modifying enzyme that might serve as a general stress marker gene (Dong et al., 2011). Arabidopsis plants overexpressing a mannose-6-phosphate reductase have increased levels of mannitol and are more tolerant to abiotic stress. Transcriptome analysis identified numerous cell wall modifying genes, which are upregulated in these plants among them xylosyltransferases involved in xyloglucan biosynthesis as well as XTH members. The authors concluded that these genes are ineeded for cell growth and cell wall strengthening as a response to the accumulating mannitol (Chan et al., 2011). Thus, many abiotic stress conditions lead to an increase in one or a few XTH genes. Expression pattern for XTH genes in Arabidopsis under such stress conditions are shown in Figure 1.

The xyloglucan-cellulose interaction cell wall model was recently challenged by the generation of a novel double-mutant *xxt1/xxt2* in Arabidopsis, which lacks classical xyloglucan due to a disruption of both xyloglucan xylosetransferases (*XXT*) genes (Cavalier et al., 2008). The mutant has a surprisingly normal phenotype considering the attributed major role of xyloglucan for the stability of the primary cell wall. Biomechanical measurements however showed a reduced stiffness. The *xxt1/xxt2* mutant has led to a rethinking of the role of xyloglucan polymers in primary cell walls. One revised model comes to the conclusion that only a minor portion of the xyloglucan, which is not accessible to XTH enzymes, is involved in cellulose interaction. Using a creep cell wall extension assay Park and Cosgrove (2012) showed that significant creep was only observed with glucanases which also cleave cellulose beside xyloglucan, suggesting that the tight connection must be modified for extension of the wall. This model however does not exclude the possibility that xyloglucan, which is at a given time point accessible for XTH, later becomes part of the inaccessible xyloglucan involved in the strong network with cellulose.

## Expansins

Expansin genes are also often transcriptionally upregulated by abiotic stress conditions. Motivated by such findings Han et al. (2012) overexpressed a wheat β-expansin (*TaEXPB23*) in tobacco. Roots of overexpressor lines developed far better under high salt conditions than wild type tobacco seedlings. It was suggested that seedlings with increased expansin expression have a higher water retention ability, though the possible mechanism as well as the potential change in the cell walls remain unclear. The overexpression of expansin A4 from rose in Arabidopsis leads to higher germination rates under salt stress, longer roots and an increase of lateral roots (Lu et al., 2013). Furthermore, the overexpressing lines are more drought tolerant and recover after the stress, whereas all wild type plants died (Dai et al., 2012). The gene was originally identified by the strong induction in rose petals undergoing dehydration. Knockout of the Arabidopsis expansin like gene *AtEXLA2* leads to plants with longer roots than the wild type under normal growth conditions but renders the roots to be more sensitive to salt stress (Abuqamar et al., 2013). This is in line with previous studies which suggest the necessity of a balanced expansin activity for normal cell growth and wall remodeling.

Rice plants stop to elongate the internodial sections under drought stress leading to stunted plants. To unravel the process Todaka et al. (2012) searched for drought regulated genes and identified the transcription factor OsPIL1 as a key regulator in this process. The gene for OsPIL1 is down-regulated under drought. They identified a number of target genes, including several expansin genes. Using a *OsEXPA4A::GUS* reporter gene construct they demonstrated a strong directly OsPil1-dependent induction of *OsEXP4A* by coexpression of the transcription factor in a rice protoplast system. Ectopic expression of OsPIL1 leads to rice plants with long internodes, whereas repression of the gene results in very short plants suggesting that the coordinated expression of expanins among other genes is mainly responsible for internodial cell length and thus for plant height.

In plants both, expansins and XTH, are present as a larger gene family with 35 members (Lee et al., 2001) for expansins and 33 members for XTH (Rose et al., 2002). Figure 1 shows the relative expression rate of the whole gene family in control shoots and roots as well as under different abiotic stress conditions. Note that not all members are represented on the ATH1-microarray chip. Only the dataset from Atgenexpress was used (Kilian et al., 2007). The heat map shows that only a few members of each gene family respond to each stress factor and it also points to different responses in shoots and roots.

**Figure 2:**
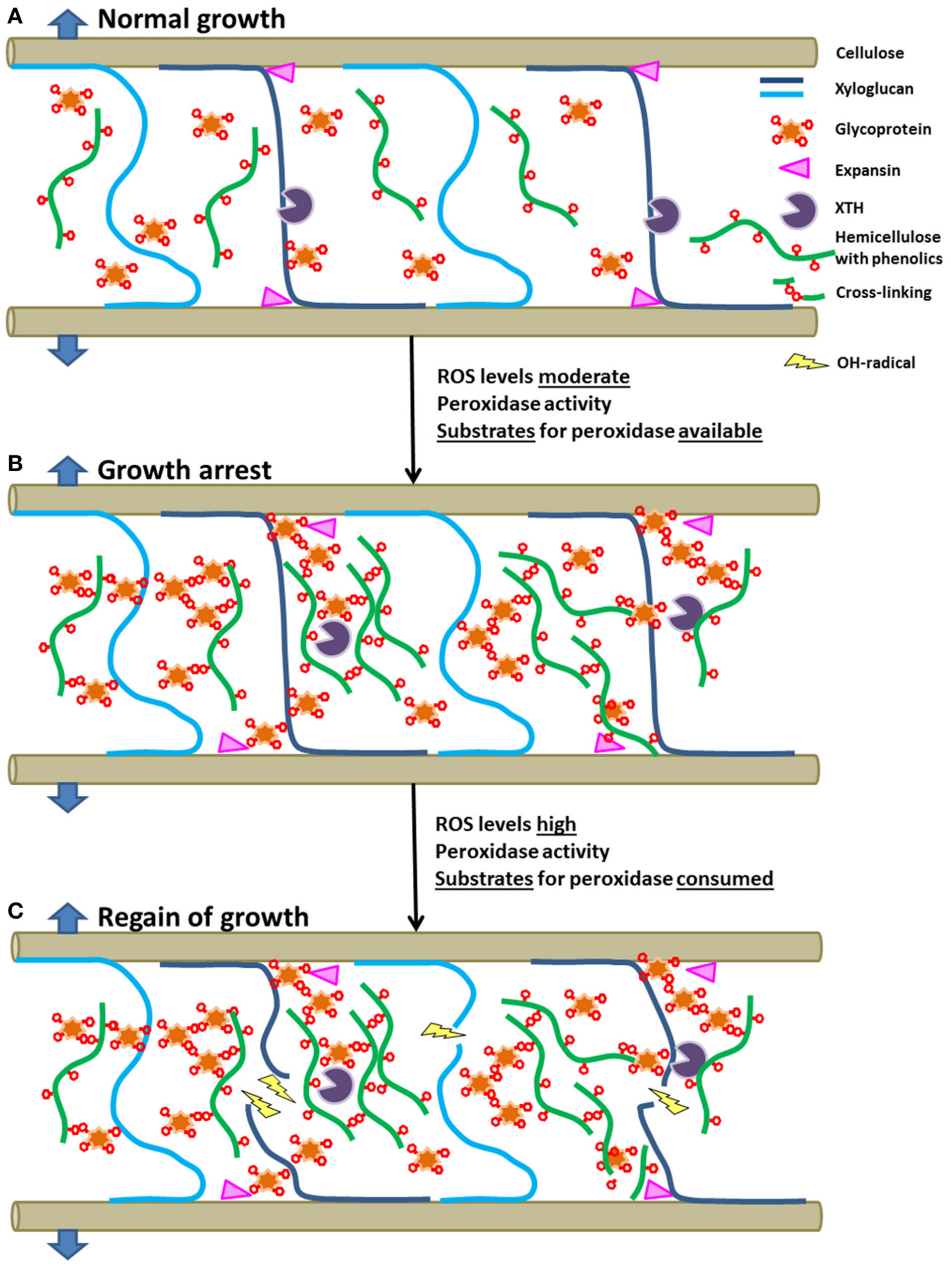
**Model of growth of cell walls in unstressed conditions (A), plants under abiotic stress showing growth arrest (B), and tolerant plants overcoming the growth arrest by using ROS-mediated cleavage of cell wall polymers (C) (see text for details)**.

Growth of plant organs under stress is apparently a conflict between stiffening of cell walls by cross-linking and loosening them by ROS, expansins and XTH. A possible simplified model for growth under abiotic stress is presented in Figure 2. The frequently observed growth arrest under abiotic stress may be caused by cross-linking of glycoproteins and phenolics esterified with hemicellulose polymers. This process requires ROS, the activity of peroxidases and substrates for the enzyme in balanced quantities. The cross-linking results in a dense network possibly preventing the undisturbed access of expansins and XTH to the xyloglucan substrate (Figure 2B). If ROS production continues and all cross-linkable substrates are used up by previous peroxidase activity, elevated ROS-levels may cause radical mediated cleavage of polymer chains (Figure 2C). This allows growth again as the OH°-radical mediated cell wall loosening process is functionally equivalent to growth in unstressed plants, in which enzymes like expansins and XTH are responsible for cell wall loosening.

## Changes in cell wall composition caused by abiotic stress

The pectins are often modified in plants exposed to drought stress. Leucci et al. (2008) compared the cell wall of two wheat cultivars differing in their tolerance toward drought stress under stress conditions. The major finding was the increase in side chains of the pectic polymers rhamnogalacturonan I and II (RGI and RGII), possibly because the pectins form hydrated gels which limit the damage to cells (Leucci et al., 2008). Furthermore, the biosynthesis of pectic polymers under drought stress was less affected in the tolerant cultivar (Piro et al., 2003). A similar study with wheat seedlings comparing a drought sensitive with a tolerant line also showed more pectins in the tolerant cultivar, especially in young seedlings (Konno et al., 2008). A comparison of the cell wall composition of root tips from two soybean cultivars showed far higher levels of pectins in the salt tolerant cultivar than in the sensitive line, suggesting that the higher pectin content is beneficial for root growth under salt stress conditions (An et al., 2014). Older plant material from two wheat cultivars with different salt tolerance was compared by Uddin et al. (2013). Unesterified uronic acids increased upon salt stress in both cultivars. However, the increase in uronic acids in the salt tolerant cultivar was slower and stopped at a lower level compared to the sensitive line. The method did not allow discriminating between galacturonic acid from pectins and glucuronic acid from the arabinoxylans. Thus, the exact change in the cell wall remains to be determined.

Rakszegi et al. (2014) compared the cell wall in three wheat cultivars, all tolerant to heat and drought, though at a different level. The major finding was the increase in the dietary fiber arabinoxylan in all cultivars under both stress conditions.

Frost tolerance of different Miscanthus genotypes is associated with compositional changes in the cell wall. The amount of mixed-linked glycans strongly increases in all cultivars after cold acclimatization. The sum of galacturonic and glucuronic acid however increases in frost sensitive lines but decreases in the tolerant one. All lines respond with a dramatic increase of cinnamyl alcohol dehydrogenase after cold acclimatization suggesting that enhanced lignification is associated with cold temperature treatment (Domon et al., 2013). The changes in lignin content and composition caused by various stress factors was recently reviewed (Magalhaes Silva Moura et al., 2010).

### Are resurrection plants a model for cell wall adaption under limited water availability?

Resurrection plants survive dehydration of their vegetative tissues to air dry state for extended periods and recover full metabolic competence upon rehydration (Rascio and La Rocca, 2005; Moore et al., 2009; Farrant and Moore, 2011; Gechev et al., 2012; Dinakar and Bartels, 2013). The water loss may be higher than 90% and is associated with a drastic shrinkage of cell volume. The physiological changes have been studied intensively in some model resurrection plants like *Craterostigma plantagineum* or *Xerophyta viscosa*. Analysis of cell walls from resurrection plants either in the hydrated or the desiccated stage revealed species specific differences (Moore et al., 2013). Upon desiccation several species have increased levels of arabinose, which are found in fractions associated with pectins and arabino-galactan-proteins. High arabinan levels are also present in the cell wall of guard cells from *Commelina communis* (Jones et al., 2003), where they are believed to be responsible for the maintenance of fluidity within the pectin network of the cell wall. A study with intact RG-I from potato clearly showed by NMR techniques, that arabinan side chains are hydrated faster than galactan side chains (Larsen et al., 2011). Therefore, a high arabinan amount will readily rehydrate cell walls after drought.

Cell walls of seeds dry out during maturation and a number of seed cell walls also contain higher arabinan levels than the vegetative tissue (Mosele et al., 2011; Gribaa et al., 2013). During germination the arabinan is largely metabolized and serves as a precursor for the synthesis of new wall polymers or arabino-galactan proteins upon activation to UDP-arabinose (Gomez et al., 2009). The high arabinan levels in seed cell walls as well as in desiccated resurrection plants has led to two conclusions. First of all, the arabinans likely fulfill a function as pectic plasticizers to keep the cell wall flexible under abiotic stress (Moore et al., 2013). Second, the mechanism of high arabinan in cell walls from drying tissue is an invention of seeds which was later adopted by resurrection plants. Whether the plasticizer hypothesis fits for a broad variety of plants needs to be addressed in future. An increase in arabinose for instance was not found in the resurrections plants *Xerophyta viscosa* or *X. humilis* (Moore et al., 2013). Arabidopsis *mur-4* mutants in the enzyme UDP-xylose epimerase, which provides UDP-arabinose as a precursor for polymer synthesis, have extremely low levels of arabinose in their cell walls. The reduction was investigated in different tissues showing a 50–75% decrease in arabinose (Burget and Reiter, 1999). However, *mur-4* mutants show no visible changes in the phenotype, have viable seeds and a normal seed set (Burget et al., 2003), which is not expected if the arabinan plasticizer role is critical for desiccating cell walls during seed maturation.

## Cell wall integrity control

The perception of abiotic stress in the cell wall likely involves members of different receptor-like kinases, comprising a very large family of integral plasma membrane proteins. These receptor-like kinases are believed to perceive changes of the environment in the extracellular space and transmit their signal into the cell, using either second messengers, reactive oxygen species, interfering with abscisic acid signaling or by phosphorylating transcription factors or other unidentified signaling proteins (Lindner et al., 2012; Wolf et al., 2012; Osakabe et al., 2013; Doblin et al., 2014). Many of the genes for receptor-like kinases are induced by abiotic stress itself, thereby amplifying the signal for the necessary stress adaption response (Lindner et al., 2012). A long known example for this is RPK1 from Arabidopsis. Constitutive expression of RPK1 causes an upregulation of a number of stress induced genes and results in an enhanced abiotic stress tolerance (Osakabe et al., 2010). Furthermore, target genes of RPK1 are upregulated in a similar fashion by overexpression of RPK1 as observed by applying stress factors like drought, salt, cold, or heat. The same RPK1 was recently identified as a major determinant for the regeneration frequency of shoots from Arabidopsis calli, a process that is unrelated to abiotic stress but may be linked to abscisic acid signaling (Motte et al., 2014). This raises the question whether RPK1 is a primary receptor for abiotic stress or whether it is involved in transducing the stress signal. Unfortunately, the mechanism by which receptor-like kinases perceive the environmental stress remains to be elucidated. A second group of cell wall integrity receptors is the CrRLK1 family originally identified in *Catharanthus roseus*. The well-studied members of this group in Arabidopsis include FERONIA (FER) (Huck et al., 2003), THESEUS (THE1) (Hematy et al., 2007) and HERKULES (HERK1, HERK2) (Guo et al., 2009). FER is involved in controlling pollen tube growth and finally rupture during fertilization, a process requiring ROS in particular OH° radicals (Duan et al., 2014). *fer*-mutants show a reduced cell length in hypocotyls (Deslauriers and Larsen, 2010). Reduced cell elongation phenotypes were also found for mutants in *the1* and *herk1* when both mutants were combined. Though CrRLK are primarily involved in controlling cell wall integrity under normal physiological conditions, they might provide a framework for the control of cell walls under abiotic stress. Common is the biochemical activation of NADPH-oxidases of the RBOH-family as well as the induction of peroxidase genes. For the formation of localized lignin deposition in the Casparian stripe an interaction of peroxidases and superoxide producing NADPH-oxidases was shown recently (Lee et al., 2013).

## Boron stress of plants

Boron is an essential plant micronutrient required for cross-linking side chains of pectin RGII. It forms diesters between apiose residues (Ishii et al., 1999), the first sugar of some RGII side chains and thereby stabilizes the pectic network and the cell wall itself. Boron deficiency in crop plants is associated with an increase in thickness of the cell wall often referred to as the swollen cell wall phenotype. Thus, the increase in thickness is not caused by deposition of more carbohydrate polymers but by an increase of the pore size of the cell wall (Fleischer et al., 1999). A similar phenotype is observed in plants which have a reduced availability of UDP-apiose and thus less side chains in RGII, though the boron availability is normal (Ahn et al., 2006; Reboul et al., 2011).

Plants grown under limiting boron concentrations are frequently observed on soils with high rain falls, which leach out the boric acid released from rocks. They exhibit pleiotropic phenotypes like inhibition of growth, damage in xylem vessels, and disturbance of plasma membrane transport processes covered by recent reviews (Camacho-Cristobal et al., 2008; Wimmer and Eichert, 2013). In an attempt to dissect the response Koshiba et al. (2009) mimicked boron deprivation in fast growing tobacco BY2 cell cultures by changing the culture medium. Within 12 h of boron deficiency the first cells undergo programmed cell death accompanied by the production of ROS and lipid break down products (Koshiba et al., 2009). This suggests that the drastic change in cell wall architecture activates the cell wall quality control system to a level which promotes programmed cell death as a response. Similarly, virus mediated silencing of UDP-apiose/UDP-xylose synthases in tobacco cause a strong increase in ROS and cell death in tobacco leaves (Ahn et al., 2006). Boron needs to be present in an optimal concentration because too high levels of boron are phytotoxic. This observation was recently addressed by the finding that normal boron levels also cross-link glycosylinositol phosphorylceramides of the plasma membrane with arabinogalactan proteins of the cell wall, thereby attaching the membrane to the cell wall (Voxeur and Fry, 2014). High concentrations of boron disrupt this interaction which may explain the phytotoxicity of high levels of boron. The many phenotypes observed with either too low or too high concentrations of boron have stimulated a discussion that proposes a signaling function for boron (Goldbach and Wimmer, 2007; Gonzalez-Fontes et al., 2008, 2014). Plants grown under boron deficiency will likely activate a part of the cell wall integrity control system, which might then activate signaling pathways indirectly observed as boron mediated signaling.

## Conclusions

The cell wall is clearly affected by many abiotic stress conditions. A common plant response is the production of ROS and an increase in the activity of peroxidases, XTH and expansins. Most data come from transcriptome analysis rather than biochemical experiments. The balance between the formation of ROS and the transcriptional changes for cell wall remodeling enzymes seems to be important for the outcome, either growth arrest in salt and drought sensitive cultivars or a continuation of (preferentially root) growth, though with a reduced rate. Surprisingly little is known about changes in the cell wall itself, a challenge for a better understanding of abiotic stress tolerance.

### Conflict of interest statement

The author declares that the research was conducted in the absence of any commercial or financial relationships that could be construed as a potential conflict of interest.
